# Opening Address to the American Medical Association

**Published:** 1883-07

**Authors:** John M. Atlee

**Affiliations:** President of the American Medical Association, at its stated session, Cleveland


CHICAGO MEDICAL

JOURNAL & EXAMINER.

VOL. XLVII.—JULY, 1883.—NO. 1.

### Original Lectures.

ARTICLE I.

OPENING ADDRESS. By JOHN M. ATLEE, M.D., LL D., President of the American Medical Association, at its stated session, Cleveland, June 5th.

*Gentlemen of the American Medical Association:* Permit me to express my feelings of gratitude for the unexpected honor conferred upon me at the last meeting of the association, and to cherish the hope that in fulfilling the duties of this responsible position, I may be sustained by your cordial co-operation. We meet here to engage earnestly in furthering the interests and objects of the medical profession. We have come together from all parts of our broad country, charged with these great responsibilities. It is fitting to express here deep regret at the absence from our councils of delegates from the Medical Society of New York. Let us hope that this absence may be only temporary, and that at the next meeting every state may be represented. As specialties are so much in favor at the present time, I have thought it well, though far from favoring them on ordinary occasions, to bring prominently forward, in my address to-day, my own rare specialty, namely, the having been a graduate of sixty-three years' standing. Instead, therefore, of calling your attention to the more strictly scientific subjects that are so generally considered upon such an occasion as this, it has occurred to me that some reminiscences of my early medical life might not be wholly unacceptable, or devoid of interest and instruction.

When I began my medical studies, in 1815, there were but few medical colleges in the country—the medical department of the University of Pennsylvania, the College of Physicians and Surgeons of New York, and the colleges at Baltimore, Harvard, New Haven, and Lexington, Ky. The University of Pennsylvania was the leading institution, to which students from all parts of the country came. The facilities for clinical instruction at the University were confined to the Pennsylvania Hospital and the Philadelphia Almshouse; but of these lectures and the distinguished clinical teachers I shall speak again. Having had no opportunities for studying practical anatomy before matriculation at the University of Pennsylvania, I devoted myself more particularly to that branch in my first course of lectures, 1817–18. The chair was then filled by Dr. Caspar Wistar, one of the most able and accomplished teachers of anatomy which this country has produced. His amiable deportment and kind treatment of students made an impression upon me which I shall never forget, and after the lapse of more than sixty-five years the thought of him kindles in my breast emotions of genuine pleasure. As I remember him, he was of medium stature, apparently about sixty years of age, and so impressive was his teaching of anatomy up to the time of his death, which occurred very suddenly, in January, 1818, that his words remain with me yet. He was certainly a man of great personal magnetism, extremely courteous in his manners, and gentle in disposition; he was always ready to converse with the students and help them in their difficulties. It is no wonder that he was greatly beloved by the students. The announcement of his sudden death from disease of the heart, on the night after he delivered his last lecture, produced a shock among the students that I shall never forget.

Just here I may appropriately allude to the foundation of a social institution, long known in Philadelphia as "the Wistar Parties." Dr. Wistar had been in the habit of inviting to his house, on Saturday evening, men of learning and distinction, both citizens and strangers. The ability and social qualities of the professors of the University of Pennsylvania and of the eminent medical men of Philadelphia, caused always the presence of a large infusion of medical science in the composition of his parties. After his death, these gatherings were revived and continued by his friends, and they were still known as "Wistar Parties" in honor of their founder. In this way originated the celebrated social gatherings which occupied so important a share in the social annals of Philadelphia. I remember my gratification when young at meeting some distinguished gentleman from abroad, and many no less distinguished from our own country.

The course of lectures on anatomy, interrupted by the death of Dr. Wistar, was subsequently finished by Dr. John Syng Dorsey, a favorite nephew of Dr. Physick. He completed the course with credit, and was subsequently elected to fill that chair. Unfortunately, he also died after a very short illness, after delivering his introductory lecture, within a week after the beginning of the term. It was a great loss to the University, and a very severe blow to Dr. Physick, one from which he never recovered. At this period there was no American work on anatomy; but about this time Dr. Wistar's Anatomy was published, and adopted as a text-book. It was received with great favor, even with enthusiasm by the students. The assistants to the professor of anatomy at this period were Drs. William E. Horner and Hugh L. Hodge, afterwards highly distinguished in their respective branches, anatomy and midwifery.

Dr. John Redmond Coxe was the professor of chemistry in the winter of 1817-18, a grandson of Dr. John Redmond, one of the leading physicians of Philadephia in his day, and first president of the College of Physicians. Dr. Coxe had the reputation of being one of the most diligent students in Philadelphia. He was very careful in his experiments, and in lecturing was very punctual in filling the whole of the hour allotted to him. The chair of midwifery, during my first course, was filled by Dr. Thomas

C. James, a very modest and agreeable gentleman of Quaker origin. He had such a sense of delicacy that he could not bring himself to lecture on the female organs of generation, but entrusted this part of his course to Dr. Horner. Although a graduate of the University of Pennsylvania, he subsequently became a pupil of Dr. Denman, of London, whose work on midwifery, together with that of Burns, and Dr. Dewees' translation of Baudelocque, constituted the principal works on that subject. Dr. James, after Denman, was a strong advocate for the short forceps.

Dr. Nathaniel Chapman, at this time, and for many years afterwards, filled the chair of the institutes and practice of medicine. He was a most eloquent and impressive lecturer, and the idol and tried friend and benefactor of the student. He was, moreover, a man of very marked ability, eloquence, and great social qualities. Having to teach the institutes, as well as the practice of medicine, it required two courses of lectures to complete the subject. The physiology of that day was very different from that of the present. The microscope had hardly begun to be applied to the study of anatomy, and so little did Dr. Chapman appreciate it, that it was a standing joke with him to quote old Leuwenhoeck as having discovered with his microscope "twenty thousand devils playing upon the point of a needle," thus foreshadowing some of the most remarkable discoveries of the present day, especially disease germs. Professor Chapman was thoroughly posted in the departments which he taught, at that time, although they have advanced wonderfully since his day. He was a man of very imposing presence rather above the medium height, always neat in his dress, perfectly well-bred, and always obliging and polite to the students. I believe that he did more for the advancement of medicine in his day than any other person with whom I was acquainted. He established a school called Chapman's Institute, for the benefit of his private students, of whom he always had thirty or forty, and other students who chose to attend. The building was in the rear of his house with a private entrance, and he employed, as teachers of his classes, gentlemen who afterwards became eminent professors at the University and at the Jefferson Medical College, among whom may be mentioned Professor William P. Dewees, Hugh L. Hodge, and John K. Mitchell.

Last, but not least, among the Faculty of that day, was Dr. Philip Syng Physick, the great American surgeon, who that winter, 1817-1818, delivered his last course of lectures on surgery. A pupil of John Hunter, he taught the doctrines of that great man. As I recall his course of lectures, it seems to me that he was one of the most impressive teachers that I have ever listened to. Dr. Physick was remarkable for great attention to details, and in his operations upon the cadaver, he carefully observed all the rules for operating upon the living body. He also recapitulated the lecture of the preceding day before going on with his subject, by questioning the students who occupied the first two rows of seats in the amphitheater. I may refer to one incident which may illustrate his method and his carefulness. On one occasion he stumped the whole class. He had been lecturing on lithotomy the preceding day, and he put the question to the first student, "What instruments should be provided for the operation?" The answer appeared to have been correctly given, but he was not satisfied. The question was repeated to the next student, and finally to the whole class, with the same result. Dr. Physick then said it was "a pin, gentlemen, a pin," that was needed to complete the list. This showed his precision, and impressed upon us the necessity of taking care never to go to an operation without the minutest preparation.

Dr. Physick was a man of medium height, with very regular features. His face at that time was pale, as if he suffered from delicate health. He was of very abstemious habits. I remember on one occasion, at a party given at his house, when the servant brought in a tray with wine, I was standing beside Dr. Chapman, when I placed my hand upon a decanter, as I supposed, of wine; Dr. Chapman touched my elbow, and told me not to take that; I filled the glass from another bottle, and afterward asked the doctor why he had checked me. He said the first was simply colored water that Dr. Physick had provided for his own use.

In speaking of Dr. Physick's teaching, I should also say that he always lectured extemporaneously, the didactic lectures on inflammation being read by Dr. Dorsey, his nephew. Dr. Physick was dignified in his deportment, and eminently grave; we rarely saw a smile upon his face. His usual dress in the lecture-room was a blue coat with metal buttons, white vest, and drab pantaloons. He was remarkably staid and reserved in his manner, and was always regarded with reverence and great respect by the students. He never indulged in any flights of imagination, and was purely a practical lecturer who brought his knowledge from the stores of his large personal experience.

One of his favorite precepts was to insist upon great attention to diet after surgical operations. I may mention this anecdote. In one of his lectures he spoke of a very important surgical operation, and said that there was a necessity for attention to absolute diet. The next day, in recapitulating, he asked a student what was meant by absolute diet. The student said "toast or barley water." "Will any gentleman tell me what is meant by absolute diet?" appealing to the whole class. There was no reply. "Water, gentlemen, water." A precept I have never forgotten, and which, I think, is not sufficiently observed at the present day after important surgical operations.

The clinical teaching of that day was not given at the medical college, as it now is, but at the Pennsylvania hospital, and the Philadelphia almshouse, then in the city; each institution affording an excellent school of instruction to the students. As the clinical hours were the same at both institutions, I chose the almshouse, as affording a larger field.

Among the clinical teachers of that day, very few were superior to Dr. Joseph Parrish, who had been a pupil of Dr. Wistar. He was a man of most amiable character, thoroughly devoted to the advancement of the profession, having large classes of private students every year, to whom he lectured, and for whom he also provided able assistants to aid in teaching. One of these was the late Dr. George B. Wood. Dr. Parrish was a man of warm sympathies, and he testified to his benevolence by the manner in which he conducted his clinics. Let me give you an illustration. A poor weather-beaten sailor was brought to the almshouse suffering very much from rheumatism. Dr. Parrish ordered the man to be clothed in flannel, and have a bottle of porter daily. On the next clinical day, Dr. Parrish, on inquiring, found that neither had been attended to. He repeated the order, with a mild rebuke to the steward. At the next visit, three days afterward, finding that his previous orders had been disobeyed, he called for the steward, and remained at the bedside of the patient until the order was fulfilled.

With regard to the treatment of that day, I shall say little; the text-books then studied fairly present it to you. Would that I could speak more satisfactorily of the treatment of the insane as I remember it. They were generally confined in the basement of the almshouse in small cells, some with manacles, others with chains, seldom had they access to fresh air, and often they had nothing but loose straw for their bedding. This unhappy and inhuman state of things continued until Pinel and Esquirol established a course of treatment more consistent with the dictates of science and humanity. In a recent visit to the State Lunatic Hospital at Harrisburg, Pa., of which I am a trustee, not one of the four hundred inmates was the subject of mechanical restraint.

At that time the resident physicians at the almshouse were not graduates in medicine, but last-course students, who fulfilled their duties while preparing for graduation. The requirements for graduation were attendance upon two full courses of lectures, of four months each, a written thesis on some medical subject, attendance at the hospital or almshouse, and an oral examination in the presence of the whole faculty.

Many of the elderly gentlemen present to-day must have heard of the much-dreaded "green-box." During the time of Drs. Rush and Barton it was reported that favoritism was shown to their respective students, and the same was said of the students of Drs. Chapman and Dorsey. To obviate this, or the appearance of it, a large green screen was placed across one corner of the room, having a door behind, through which the candidate entered, and here underwent his examination, unknown to any one but the dean of the faculty. This mode of examination was adhered to until after the death of Dr. Dorsey, when it was optional with the student to go into the green-box or present himself openly before the faculty. Some ten or twelve candidates had such a terror of the green-box that they went to New York, where they obtained the degree of M.D. by undergoing an examination and paying the graduating fee.

As to my own experience in the green-box, it has been always an amusing recollection as to the terror with which it inspired me, and my intense relief at finding the dreaded ordeal consist of nothing more than a question or two as to my knowledge of Glauber's salts.

It was the time of calomel and the lancet. With regard to the one, I need not speak; but of the latter I feel assured that the almost total disuse into which it has fallen has cost many valuable lives. From a very large experience in its use, I am satisfied, fully satisfied, that if we depended more on the early use of the lancet in the congestive and inflammatory states of many diseases, our practice would be more successful than it now is. At the present time there is too exclusive reliance upon medicines affecting the nervous and vascular systems, which act with less efficiency and are less prompt. It is, in my opinion, a very important subject, and I feel assured that ere long the lancet will be more freely used than it is now. In the congestive chills preceding inflammatory diseases, and in the cold stages of intermittents, I have frequently broken up the paroxysm, and relieved the patient by the lancet alone.

In the class of 1817–18, there were many men who afterwards became distinguished in their respective departments. Time will not permit me to enumerate them all.

In my day, previous to the establishment of medical societies throughout the country, and the organization of the American Medical Association, and the general adoption of the Code of Ethics, I saw many disastrous effects from the want of brotherly consideration and kindness. The medical men of that day were often in difficulties; patients would be taken from one physician to another without ceremony; and so great was the jealousy existing between them, that for more than twenty years after my graduation, it was impossible to form a medical society in my native city and county, because there were so many aspirants for the honors. Here let me speak of some of the difficulties I had to encounter in my early professional life. Instead of being taken by the hand by the older physicians, every obstacle was thrown in my path—consultations were refused, and the treatment of my patients unfavorably criticized.

By the establishment of medical societies and the adoption of the Code of Ethics, a wonderful change has been effected. We now feel it our duty to sustain our younger brethren, to treat them with courtesy and kindness, to save them from their errors, and encourage them in all their good work. Had the adoption of the Code of Ethics no other result than this, it would have been an invaluable blessing to the profession. But it has accomplished more. It has put the seal of condemnation upon all "isms," and developed an *esprit de corps* that has enlarged the boundaries of our science, and greatly increased the usefulness and social standing of the profession.

Now, gentlemen, before concluding, let me state that, being aware that reports and papers upon every important topics connected with the different departments of medicine will be presented by the chairman of the sections, and by individual members, I have not entered upon the discussion of any subject, either medical or surgical.

Our meetings are for the purpose of promoting social intercourse, as well as for the advancement of medical science; but we should devote sufficient time to the discussion of the various subjects presented to us, and not allow them to be too greatly interfered with by social entertainments.

Above all things, ever strive to maintain the honor and dignity of the profession. Let no selfish or mercenary consideration deter you from observing the laws laid down in our noble Code of Medical Ethics. Cultivate friendly relations with your local medical brethren, more particularly the younger: and regulate your intercourse with all men in such a way as to cast no stain upon the honor of the profession, which is in your keeping.

One word more, and I have done, and I say it chiefly as a word of encouragement to the younger among you. At the close of a long life, one devoted unreservedly to the study and practice of medicine, I will say that notwithstanding its uncertainties, its fatigues, its anxieties, its bitter disappointments, I am completely satisfied that in no other career can a man more fully accomplish his whole duty to God and to his fellow men; so that when life here is ended, it can truly said of him as—be it said with all reverence—was said of him whom we should all imitate, *pertransivit benefaciendo*—he went about doing good. Trusting that our proceedings may be both harmonious and profitable to us all; and thanking you again for the honor you have conferred upon me, I sincerely hope that the recollections we shall carry home with us will be both agreeable and lasting.

---

A LARGE section of the senior class of the Woman's Medical College paid a visit to the county Insane Asylum at Jefferson on the 19th of May, and were treated with every courtesy by the able Superintendent, Dr. J. C. Spray, and his gentlemanly assistant, Dr. Alex. Thuemmln, who kindly escorted them through all the wards, and pointed out many items of interest to the medical student. After an hour spent in the wards, the party found themselves in the amusement hall, where Professor D. R. Brower held a most interesting clinic, presenting typical cases of the various forms of insanity at present in the institution.

A visit was then made to the Cook County Infirmary, near the asylum, under the care of Dr. A. W. Hagenbach, who did the honors of the institution in hospitable style, presenting a number of interesting types of paralysis and chronic surgical cases. Among the latter was one lately sent out from the homœopathic side of Cook County Hospital, accompanied with the ambiguous diagnosis of *pain*. The doctor resorted to the old-fogy treatment of relieving the left pleural cavity of thirty-one ounces of serum, with quite happy effect upon the condition of the patient, subsequently relieving him by the same method of a similar quantity.

Though notice had not been received of the intended visit, both the institutions presented the appearance of "visitor's day;" floors, woodwork and bedding spotless; the attendants quiet and orderly; the patients under thorough control. The gentlemen in charge assert this to be their everyday appearance, and are ready for inspection by the profession at any time. There are at present over eleven hundred patients in the two institutions.

